# Association of breakfast intake with obesity, dietary and physical activity behavior among urban school-aged adolescents in Delhi, India: results of a cross-sectional study

**DOI:** 10.1186/1471-2458-12-881

**Published:** 2012-10-17

**Authors:** Monika Arora, Gaurang P Nazar, Vinay K Gupta, Cheryl L Perry, K Srinath Reddy, Melissa H Stigler

**Affiliations:** 1 Health Related Information Dissemination Amongst Youth (HRIDAY), Safdarjung Development Area, New Delhi 110016, India; 2 Public Health Foundation of India (PHFI), 4/2 Sirifort Institutional Area, August Kranti Marg, New Delhi 110016, India; 3 UTHealth, School of Public Health, University of Texas, Austin Regional Campus Michael & Susan Dell Center for Healthy Living, 1616 Guadalupe, Austin, TX 78701, USA

**Keywords:** Breakfast, Obesity, Adolescent, Diet, Physical activity, Behavior

## Abstract

**Background:**

In developed countries, regular breakfast consumption is inversely associated with excess weight and directly associated with better dietary and improved physical activity behaviors. Our objective was to describe the frequency of breakfast consumption among school-going adolescents in Delhi and evaluate its association with overweight and obesity as well as other dietary, physical activity, and sedentary behaviors.

**Methods:**

*Design:* Cross-sectional study. *Setting:* Eight schools (Private and Government) of Delhi in the year 2006. *Participants:* 1814 students from 8^th^ and 10^th^ grades; response rate was 87.2%; 55% were 8^th^ graders, 60% were boys and 52% attended Private schools. *Main outcome measures:* Body mass index, self-reported breakfast consumption, diet and physical activity related behaviors, and psychosocial factors. *Data analysis:* Mixed effects regression models were employed, adjusting for age, gender, grade level and school type (SES).

**Results:**

Significantly more Government school (lower SES) students consumed breakfast daily as compared to Private school (higher SES) students (73.8% vs. 66.3%; *p<0.01*). More 8^th^ graders consumed breakfast daily vs.10^th^ graders (72.3% vs. 67.0%; *p<0.05*). A dose–response relationship was observed such that overall prevalence of overweight and obesity among adolescents who consumed breakfast daily (14.6%) was significantly lower vs. those who only sometimes (15.2%) or never (22.9%) consumed breakfast (*p<0.05 for trend*). This relationship was statistically significant for boys (15.4 % vs. 16.5% vs. 26.0; *p<0.05 for trend*) but not for girls. Intake of dairy products, fruits and vegetables was 5.5 (95% CI 2.4-12.5), 1.7 (95% CI 1.1-2.5) and 2.2 (95% CI 1.3-3.5) times higher among those who consumed breakfast daily vs. those who never consumed breakfast. Breakfast consumption was associated with greater physical activity vs. those who never consumed breakfast. Positive values and beliefs about healthy eating; body image satisfaction; and positive peer and parental influence were positively associated with daily breakfast consumption, while depression was negatively associated.

**Conclusion:**

Daily breakfast consumption is associated with less overweight and obesity and with healthier dietary- and physical activity-related behaviors among urban Indian students. Although prospective studies should confirm the present results, intervention programs to prevent or treat childhood obesity in India should consider emphasizing regular breakfast consumption.

## Background

It is estimated that 30% of obesity begins in childhood [1], and about 50-80% of obese children become obese adults [2]. Although this epidemic is well-described in the developed world, far fewer studies have been conducted in developing countries, like India, where the prevalence is escalating [3]. A review of childhood obesity prevalence studies in India revealed a large degree of variation in the prevalence of overweight (8.5-29.0%) and obesity (1.5-7.4%) among school-aged youth, with the highest prevalence among urban youth and youth of higher socio-economic status (SES) [4]. In a recent publication from our research team for this study, the combined prevalence of overweight and obesity among school-going adolescents (12–18 years) in Delhi was shown to be 16.6% [5]. The prevalence was up to seven fold higher among Private school students as compared to those in Government schools (26.6% *vs.* 3.9%, *P*<0.001) [6]. Thus, in higher SES classes in India, the prevalence of overweight and obesity among school-aged youth is now on par with the prevalence in many countries of the developed world [4]. Ultimately, the impact of this epidemic in India may be larger than that in the developed world, as the negative health consequences of obesity, like diabetes, occur up to a decade earlier in India than they do in the West [7], and at comparatively lower BMI values [8]. The need for preventive interventions is great and this should be driven by etiologic research, which is lacking in this context at present.

The etiology of childhood obesity is complex and multi-factorial and in the developed world, includes a myriad of behavioral, intra-personal, and social-environmental risk and protective factors [9-11]. Notable among these is the consumption of breakfast. Many studies undertaken in developed countries [12-16], and some developing nations, like Iran [17], suggest that the frequency of breakfast consumption is inversely associated with Body Mass Index (BMI) among school-going children and adolescents. A systematic review of cross-sectional and longitudinal studies from Europe suggests that infrequent or never breakfast consumers are at higher risk of being overweight and obese [18]. The protective effect of breakfast consumption on healthier weight status may be conferred through improvements in appetite control and nutritional profiles [19-21]. More recently however, another systematic review suggested that although a large number of cross-sectional studies demonstrate an inverse association between regular breakfast consumption and excess weight among children and adolescents, several longitudinal studies fail to demonstrate this relationship, after good adjustment for potential confounders like physical activity, energy intake and other dietary and sedentary behaviors [22]. Other studies have shown that regular consumption of breakfast is associated with higher physical activity levels [16,23].

Selected psychosocial risk factors have been shown to predict obesogenic patterns of dietary intake and physical activity in the West [24-26]. These measures include intra-personal factors (e.g., values, beliefs, satisfaction with body image, depression, eating more than usual) and social-environmental factors (e.g., peer influence and parent influence), based on a social ecological model of the etiology of childhood obesity [27]. In studies from developed countries, positive attitudes towards breakfast consumption have been shown to be associated with more frequent breakfast consumption, as have been the positive influences of parents and peers [28-30]. Additional benefits of regular breakfast consumption include improved cognitive function and academic achievement [31-33], making this behavior especially worthy of study among school children.

Though breakfast is considered to be the first and most important meal of the day, very few research studies in India have focused on this potentially protective behavioral determinant. According to a study undertaken in Andhra Pradesh, India, more than half of school children there skip breakfast on at least some days of the week [34]. Breakfast skippers in India have been shown to have inadequate intakes of key nutrients that could not be made up at later meals, including macro-nutrients like energy and protein and micro-nutrients like vitamins A, C, and iron [34,35]. To date, no published studies have explored whether breakfast consumption is related to overweight status among school children in India. In addition, psychosocial correlates of breakfast consumption by children and adolescents are hitherto unexplored in the Indian context. Through this study, we aimed to (a) describe the frequency of breakfast consumption among school-going adolescents in Delhi, India; (b) evaluate its association with obesity and overweight; and (c) describe its relationship to other dietary, physical activity, and sedentary behaviors and related psychosocial factors.

## Methods

### Study design and setting

This research was built on the infrastructure of an existing research study called ‘Mobilizing Youth for Tobacco-Related Initiatives in India (Project MYTRI)’. Project MYTRI was a nested cohort, group-randomized trial, with a long-term goal to prevent and reduce tobacco use among young people in the 6^th^ to 10^th^ grades in Delhi and Chennai, over the years 2004–2006. In 2004, thirty two schools in Delhi (n=16) and Chennai (n=16) were recruited using convenience sampling to participate, matched on type of school [Private (high SES) vs. Government (low SES); Co-educational vs. Boys-only vs.Girls-only], and randomly assigned to receive a two year school-based tobacco prevention program (16 intervention schools in Delhi and Chennai) or serve as control (16 control schools in Delhi and Chennai). Additional details about Project MYTRI are published elsewhere [36-38]. The data for the current study was collected along with MYTRI’s endline survey in 2006 in eight intervention schools of Delhi; no dietary interventions, including breakfast interventions, had been conducted in these schools. These eight schools included four Private co- educational schools, two Government co-educational schools, one Government boys only school and one Government girls only school. Although not a random sample, these schools were selected because they were representative of the range of types of schools in these urban cities, including Government (low-to-middle income), Private (middle-to upper income), girls-only, boys-only, and co-educational schools. The present study is cross-sectional by design.

### Participants

All students enrolled in 8^th^ and 10^th^ grades in the eight schools of Delhi described above were eligible and were invited for participation (n=2339). Response rates for the BP survey and anthropometric measurements were 88.6% and 87.2% respectively. The final sample for analysis in this study consisted of 1814 students who participated in all data collection efforts and had complete data on the breakfast consumption question. Of these, 55% were enrolled in 8th grade (vs. 10th grade), 60% were boys (vs. girls) and 52% attended a Private (vs. Government school). The average age of the students was 14.29 years (age range 12–18 years).

Active informed consent was sought from the schools and students provided informed assent. Parents provided informed, passive consent for the study where if the parents didn’t want their children to participate in the study, they were required to return the signed refusals. Study procedures including data collection, consent forms and respective procedure and survey questionnaire were approved by ethics committee at the All India Institute of Medical Sciences (AIIMS) and Independent Ethics Committee (IEC), Mumbai as well as Institutional Review Board (IRB), University of Texas.

### Measures

#### Anthropometric measures

Heights and weights of students were measured using standardized protocols adapted from Lohman and colleagues to meet the needs specific to the Indian context [39]. For measurements, the students were asked to remove all excess clothing (e.g. sweaters, coats etc.) other than their regular school uniform, shoes, all items from pockets, watches, eye-glasses, belts, necklaces, and other jewellery. When required (e.g., for Sikhs wearing turbans), the students were also asked to adjust their hairstyle. Weight was measured to the nearest 0.1 kg using Salter Electronic Scale (model 920). Height was measured to the nearest 0.1 cm using ASCOR manual calibrated vertical rod. These measurements were used to calculate BMI [weight (kg)/height (m^2^)] of students [40]. Age and gender specific BMI cut-points were used to classify participants as underweight, normal weight, overweight or obese using the 2007 WHO growth reference for school aged children (5–19 years old) [41]. This growth reference is well-suited to the Indian context [6].

#### Behavioral-psychosocial (BP) survey

A questionnaire including measures of dietary intake, physical activity, sedentary behavior, and psychosocial risk factors was developed by adapting measures from reliable instruments that have been validated with adolescents. Surveys that were referred to included: HRIDAY-CATCH [42], Project EAT [43], and the SPAN survey [44]. This survey was extensively pilot tested by administering it to 159 students prior to the present study and conducting focus group discussions (FGDs) with 40 students in one Government (medium of instruction: Hindi) school and one Private (medium of instruction: English) school which were randomly selected in Delhi and were different from schools recruited for the main study. The primary purpose of this pilot testing was to establish the instrument’s content validity. Based on the observations of the survey administrators during the pilot testing and the feedback elicited by the students through FGDs, relevant modifications were made in the content and language used in the questionnaire for the main study. The final BP questionnaire was a 65 item, self-administered instrument in English (for Private schools) as well as Hindi (for Government schools), which assessed a range of socio-demographic, behavioral, socio-environmental, and intra-personal factors. Measures relevant to the present study are described below.

##### Breakfast consumption

The primary exposure variable was breakfast consumption (In the past week, on how many days did you eat breakfast (the first meal of the day)? This question had five response options, “never”, “1-2 times in a week”, “3-4 times in a week”, “5-6 times in a week” and “every day”. These options were further collapsed into three categories, “Never”, “Sometimes” (which included “1-2 times in a week,” “3-4 times in a week,” and “5-6 times in a week”) and “Daily” during analysis. This representation of breakfast consumption is consistent with prior studies [45].

#### Other behavioral factors

These included other dietary, physical activity and sedentary behaviors and are described in Table 1. The dietary behaviors included items such as consumption of dairy products, fruits, vegetables, fried foods and soft-drinks. Physical activity was reported in terms of mild, moderate and strenuous physical activity while the sedentary behaviors included items like duration of watching TV and videos, doing academic work, and using a computer. Dietary, physical activity and sedentary behaviors were binary coded (0/1) on a frequency cut-off value informed by prior research in developed countries and revised based on FGDs conducted in the pilot study. These cut-off values were almost similar to other studies conducted in the West [43,46-48].

**Table 1:** Self-Reported Dietary, Physical Activity and Sedentary Behaviors assessed through the BP Survey

**Variable**	**Question**
**Dietary Behaviors**	
Eat dairy products (yes/no)	Do you eat dairy foods (such as paneer [cottage cheese], butter etc.)?
Eat fruits (≥1 times/day)	In the past one year, how many times did you eat fruits (not counting fruit juice)?
Eat vegetables (≥2 times/day)	In the past one year, how many times did you eat vegetables (not counting carrots, potatoes or salad)?
Eat fried food (≥2 times/day)	In the past one year, how many times did you eat fried foods (such as samosa, pakoda, vada etc.)?
Drink soft-drinks (≥2 times/day)	How often do you drink soft drinks (such as Pepsi, Coca Cola, Mirinda, Sprite, 7Up, Limca etc.)?
**Physical Activity**	
Do mild exercise (≥1/2 hour/day)	In a usual week, how many hours do you spend doing mild exercise (little effort). Examples: walking slowly (to school, friends’ house, etc.), bowling, golf, yoga
Do moderate exercise (≥1/2 hour/day)	In a usual week, how many hours do you spend doing moderate exercise (not exhausting). Examples: walking quickly, cricket, gymnastics, slow cycling, volleyball, dancing, table-tennis, skipping, badminton
Do strenuous exercise (≥1/2 hour/day)	In a usual week, how many hours do you spend doing strenuous exercise (heart beats rapidly). Examples: cycling fast, aerobic exercise, jogging, swimming, laps, skating, tennis, soccer/football, basketball, throwball, kabaddi, kho kho, pitthu [a local game involving running]
**Sedentary Behaviors**	
Watch TV, Weekday (≥2 hours/day)	On an average weekday (Monday-Friday), during your free time, how many hours do you spend watching TV and videos?
Watch TV, Weekend (≥2 hours/day)	On an average weekend day (Saturday or Sunday), how many hours do you spend watching TV and videos?
Use Computer, Weekday (≥2 hours/day)	On an average weekday (Monday-Friday), during your free time, how many hours do you spend using a computer for net surfing, e-mailing, chatting, playing games, watching movies, etc.?
Use Computer, Weekend (≥2 hours/day)	On an average weekend day (Saturday or Sunday), how many hours do you spend using a computer for net surfing, e-mailing, chatting, playing games, watching movies, etc.?
Study School, Weekday (≥2 hours/day)	On an average weekday (Monday-Friday), during your free time, how many hours do you spend reading, studying and doing academic work, including tuitions?
Study School, Weekend (≥2 hours/day)	On an average weekend day (Saturday or Sunday), how many hours do you spend reading, studying and doing academic work, including tuitions?

#### Psychosocial risk factors

Psychosocial factors such as values, beliefs, satisfaction with body image, depression, eating more than usual, peer and parent influence were also included in the BP survey. Multiple item summative scales were created for these measures. As the items assessing the psychosocial factors were adopted from studies conducted in the developed countries [24-27], we only assessed face validity of these factors through the pilot study described above, using procedures that we have employed in India for more than 10 years now, which have resulted in psychometrically robust measures. The reliability of scales for the psychosocial risk factors in the present study population was tested by checking the internal consistency of the responses using Cronbach’s alpha. Table 2 briefly provides a description of each scale, including the Cronbach’s alpha and example of an item used to construct the scale. Scale scores were standardized before being used in the analysis (i.e. the mean of each scale was set to zero and its standard deviation to one), to ease interpretation of parameter estimates and allow for comparisons between scales. A higher score on all scales is protective for values, beliefs, satisfaction with body image, peer and parent influence. For depression and eating more than usual, a higher score shows more risk. The values of Cronbach’s alpha ranged from 0.7 – 0.9 and indicated high consistency among the items.

**Table 2:** Description of multi-item scales used to measure psychosocial risk factors (n=1814)

**Psychosocial factor**	**Items**	**Cronbach’s Alpha**	**Example of question**
Values	7	0.77	How important is being healthy for you?
Beliefs	5	0.84	How strongly do you agree with the statement that “The type of food I eat affects my health”?
Satisfaction with body image	5	0.84	How satisfied are you with your weight?
Depression	7	0.82	In the past one year how often have you been bothered about worrying too much?
Eating more than usual	7	0.77	Do you think you eat more than usual when you are studying for your exams?
peer’s influence	6	0.89	Many of my friends care about eating healthy food?
Parents’ influence	6	0.92	My parents encourage me for eating healthy food?

### Data analysis

Chi square tests were used to test the association between breakfast consumption and demographic variables such as age, gender, school type and grade. The differences in prevalence of overweight and obesity among the three groups of breakfast consumers were assessed using mixed effects regression models. Odds ratios were calculated to compare dietary and physical activity behaviors between daily, sometimes, and never breakfast consumers. The association between psychosocial factors and breakfast consumption were assessed using mixed effects linear regression models. Mixed effects regression models are appropriate for study designs like these, as students are sampled within schools [49]. School was specified as a nested random effect in all models and the models were estimated using maximum likelihood estimation methods, which are robust to departures from normality [50]. Although, there was no interaction of gender and school type in the relationship between breakfast and overweight and obesity, all analyses were completed for the entire sample and then segregated by school type and gender, to examine the magnitude of difference by these variables, given contextual differences between boys and girls and high SES and low SES youth that are typical in India. The regression models were adjusted for age, gender, grade and school type (when not segregated by these demographic variables). In the analysis of breakfast consumption with dietary and physical activity behaviors and psychosocial factors, BMI was also adjusted for. All statistical tests were two sided and considered significant at 5% level of significance. Statistical software SAS 9.1 was used for all the analyses.

## Results

### Breakfast consumption and demographic variables

Overall, out of 1814 participants, 30% consumed breakfast less than daily. Table 3 shows that age, grade and school type were significantly associated with breakfast consumption. Daily breakfast consumption was significantly lower among older students as compared to younger students (Chi-square statistic=12.09; *P=0.027),* higher among Government schools than Private schools (Chi-square statistic=14.26; *P=0.002*), and higher among 8^th^ graders than 10^th^ graders (Chi-square statistic=7.97; *P=0.019*). There was no significant difference by gender for frequency of breakfast consumption.

**Table 3:** Demographic profile of students and their association with breakfast consumption (n=1814)

**Demographic profile**	**N (%)**	**Breakfast %**			**Chi-sqaure value**	**p-value**
		**Never (n=129)**	**Intermediate (n=417)**	**Daily (n=1268)**		
**Gender**						
Boys	1094 (60.3)	7.3	22.2	70.5	1.01	0.604
Girls	720 (39.7)	6.8	24.2	69.0		
**Age**						
<=13	418 (23.1)	6.7	19.6	73.7	12.09	0.027
14	418 (23.1)	4.6	23.9	71.5		
15	443 (24.4)	7.2	22.4	70.4		
16 and above	534 (29.5)	9.4	25.5	65.2		
**School**						
Private	944 (52.0)	7.8	25.9	66.3	14.26	0.002
Government	870 (48.0)	6.3	19.4	73.8		
**Grade**						
8^th^	995 (54.9)	5.8	21.9	72.3	7.97	0.019
10^th^	819 (45.2)	8.7	24.3	67.0		

### Breakfast consumption and excess weight

Overall, a dose–response relationship was shown to exist between breakfast consumption and being overweight and obese among all students (Table 4). It was observed that the prevalence of overweight and obesity was lowest overall among those students who consumed breakfast daily (14.6%), higher among those who consumed breakfast sometimes (15.2%) and highest among never breakfast consumers (22.9%) (F-test statistic=3.25; *P=0.039 for trend*). Though this trend held for all sub-groups considered, differences were statistically significant among boys only (15.4% vs. 16.5% vs. 26.0; F-test statistic=3.16; *P=0.043*).

**Table 4:** Prevalence of overweight and obesity, by breakfast consumption

**Breakfast**	**N (%)**	**Overweight and Obese**† **% (95% CI)**				
		**Overall**	**School Type**		**Gender**	
			**Private**	**Government**	**Boys**	**Girls**
Never	129	22.9	37.8	7.3	26.0	18.5
	(7.1)	(15.5-30.2)	(24.6-50.9)	(2.1-12.6)	(16.5-35.6)	(8.3-28.8)
Sometimes	417	15.2	27.6	2.9	16.5	14.4
	(23.0)	(9.9-20.6)	(17.6-37.7)	(0.0-6.0)	(9.4-23.6)	(7.9-20.8)
Daily	1268	14.6	25.6	4.0	15.4	14.3
	(69.9)	(10.0-19.2)	(16.5-34.7)	(2.3-5.7)	(9.4-21.5)	(9.3-19.3)
F test value		3.25	2.45	1.07	3.16	0.35
P value		0.039	0.087	0.345	0.043	0.702

† Adjusted prevalence were obtained using mixed effect regression model. Gender, School type, age (when not segregated) and grade were adjusted and school was treated as random effect.

### Breakfast consumption and other dietary, physical activity, and sedentary behaviors

The association between other dietary behaviors and physical activity with breakfast consumption is shown in Table 5. Results show that daily breakfast consumers were 5.5 times more likely (95% CI: 2.4-12.5) to consume dairy products, 1.7 times more likely (95% CI: 1.1-2.5) to consume fruits at least once in a day, 2.2 times more likely (95% CI: 1.3-3.5) to consume vegetables at least twice in a day compared to those who never consumed breakfast. These relationships were particularly strong for Private school students (except for dairy product consumption which was higher in Government school students) and girls. There was no difference in the odds of engaging in these dietary behaviors between never and sometimes breakfast consumers, overall.

**Table 5:** Association of daily breakfast consumption with dietary, physical activity, and sedentary behaviours (n=1814)

**Dietary Behaviours**	**Breakfast**	**Overall**		**School Type**		**Gender**	
				**Private**	**Government**	**Boys**	**Girls**
		**Unadjusted OR**	**Adjusted OR**†	**OR**‡	**OR**‡	**OR§**	**OR§**
				**(95% CI)**	**(95% CI)**	**(95% CI)**	**(95% CI)**
Eat dairy products (yes/no)	Never	1.0	1.0	1.0	1.0	1.0	1.0
	Sometimes	1.9	2.2	1.3	**4.9**	1.2	**4.6**
		(0.8-4.4)	(0.9-5.2)	(0.4-4.1)	**(1.2-19.3)**	(0.4-3.9)	**(1.2-17.3)**
	Daily	**4.8**	**5.5**	**3.4**	**9.3**	**3.5**	**7.9**
		**(2.1-10.8)**	**(2.4-12.5)**	**(1.0-11.1)**	**(2.9-3.2)**	**(1.1-11.4)**	**(2.4-26.6)**
Eat fruits (≥1 times/day)	Never	1.0	1.0	1.0	1.0	1.0	1.0
	Sometimes	0.8	0.8	1.0	0.6	0.6	1.2
		(0.5-1.3)	(0.5-1.2)	(0.6-1.8)	(0.3-1.1)	(0.3-1.0)	(0.6-2.5)
	Daily	**1.7**	**1.7**	**2.3**	1.0	1.3	**2.2**
		**(1.1-2.4)**	**(1.1-2.5)**	**(1.3-3.9)**	(0.6-1.7)	(0.8-1.1)	**(1.2-4.2)**
Eat vegetables (≥2 times/day)	Never	1.0	1.0	1.0	1.0	1.0	1.0
	Sometimes	1.5	1.5	1.6	1.2	1.4	1.6
		(0.9-2.6)	(0.9-2.5)	(0.8-3.0)	(0.5-3.1)	(0.7-2.7)	(0.7-3.7)
	Daily	**2.2**	**2.2**	**2.2**	2.0	**2.1**	**2.2**
		**(1.3-3.5)**	**(1.3-3.5)**	**(1.2-4.1)**	(0.9-4.5)	**(1.1-3.9)**	**(1.0-4.8)**
Eat fried foods (≥1 times/day)	Never	1.0	1.0	1.0	1.0	1.0	1.0
	Sometimes	0.8	0.8	1.1	0.7	0.8	0.9
		(0.5-1.2)	(0.5-1.3)	(0.5-2.2)	(0.4-1.3)	(0.5-1.4)	(0.4-1.8)
	Daily	0.8	0.8	1.0	0.7	0.9	0.6
		(0.5-1.2)	(0.5-1.2)	(0.5-2.0)	(0.4-1.2)	(0.5-1.5)	(0.3-1.2)
Soft drink consumption (≥2 times/day)	Never	1.0	1.0	1.0	1.0	1.0	1.0
	Sometimes	**0.6**	0.6	0.7	0.6	0.6	0.6
		**(0.4-0.9)**	(0.4-1.0)	(0.4-1.3)	(0.3-1.1)	(0.4-1.1)	(0.3-1.3)
	Daily	**0.7**	0.7	0.7	0.7	0.7	0.6
		**(0.4-1.0)**	(0.5-1.1)	(0.4-1.3)	(0.4-1.2)	(0.4-1.2)	(0.3-1.2)
**Physical Activity**							
Strenuous (≥ ½ hr/day)	Never	1.0	1.0	1.0	1.0	1.0	1.0
	Sometimes	**1.9**	**1.9**	**1.9**	2.0	**2.0**	1.7
		**(1.2-2.9)**	**(1.2-3.0)**	**(1.1-3.3)**	(1.0-4.0)	**(1.1-3.5)**	(0.8-3.4)
	Daily	**1.7**	**1.7**	**1.8**	1.7	**1.7**	1.7
		**(1.2-2.5)**	**(1.2-2.6)**	**(1.1-3.0)**	(0.9-3.1)	**(1.0-2.8)**	(0.9-3.3)
Moderate (≥ ½ hr/day)	Never	1.0	1.0	1.0	1.0	1.0	1.0
	Sometimes	**2.8**	**2.8**	**2.4**	**3.2**	**3.9**	1.8
		**(1.8-4.5)**	**(1.7-4.5)**	**(1.2-4.9)**	**(1.6-6.4)**	**(2.0-7.6)**	(0.8-3.9)
	Daily	**2.6**	**2.7**	**2.9**	**2.4**	**2.8**	**2.4**
		**(1.7-3.9)**	**(1.7-4.1)**	**(1.5-5.4)**	**(1.3-4.4)**	**(1.6-4.8)**	**(1.2-5.0)**
Mild (≥ ½ hr/day)	Never	1.0	1.0	1.0	1.0	1.0	1.0
	Sometimes	**2.4**	**2.6**	**2.1**	**3.1**	**3.7**	1.5
		**(1.6-3.8)**	**(1.6-4.0)**	**(1.1-3.9)**	**(1.6-5.9)**	**(2.1-6.7)**	(0.7-3.0)
	Daily	**2.7**	**2.9**	**2.8**	**3.01**	**4.6**	1.5
		**(1.9-4.0)**	**(1.9-4.4)**	**(1.6-5.0)**	**(1.68-5.39)**	**(2.8-7.7)**	(0.8-3.0)
**Sedentary Activity**							
Watch TV, weekday (≥2 hr/day)	Never	1.0	1.0	1.0	1.0	1.0	1.0
	Sometimes	1.0	1.0	1.1	1.0	0.8	1.5
		(0.7-1.5)	(0.7-1.5)	(0.6-1.8)	(0.5-1.8)	(0.5-1.3)	(0.8-2.9)
	Daily	0.8	0.8	0.8	0.7	0.7	0.9
		(0.6-1.1)	(0.6-1.2)	(0.5-1.4)	(0.4-1.3)	(0.5-1.2)	(0.5-1.7)
Watch TV, weekend (≥2 hr/day)	Never	1.0	1.0	1.0	1.0	1.0	1.0
	Sometimes	1.7	1.7	2.1	1.3	1.8	1.5
		(1.1-2.5)	(1.1-2.5)	(1.2-1.5)	(0.7-2.5)	(1.1-3.1)	(0.8-2.8)
	Daily	1.3	1.3	1.4	1.2	1.5	1.0
		(0.9-1.8)	(0.9-1.8)	(0.9-2.4)	(0.7-2.0)	(0.9-2.5)	(0.6-1.9)
Use computer, weekday (≥2 hr/day)	Never	1.0	1.0	1.0	1.0	1.0	1.0
	Sometimes	0.9	1.0	1.0	0.9	0.8	1.4
		(0.6-1.4)	(0.6-1.5)	(0.6-1.9)	(0.4-1.7)	(0.8-4.2)	(0.6-3.2)
	Daily	0.8	0.9	0.8	1.1	0.8	1.3
		(0.5-1.2)	(0.6-1.3)	(0.4-1.3)	(0.6-2.0)	(0.7-3.1)	(0.6-2.7)
Use computer, weekend (≥2 hr/day)	Never	1.0	1.0	1.0	1.0	1.0	1.0
	Sometimes	1.1	1.1	0.9	0.8	0.8	1.9
		(0.7-1.6)	(0.7-1.7)	(0.5-1.9)	(0.4-1.4)	(0.5-1.4)	(0.8-4.2)
	Daily	1.0	1.0	1.2	0.7	0.9	1.4
		(0.7-1.5)	(0.7-1.6)	(0.7-2.3)	(0.4-1.2)	(0.6-1.5)	(0.7-3.1)
Study school, weekday (≥ 2 hr/day)	Never	1.0	1.0	1.0	1.0	1.0	1.0
	Sometimes	1.2	1.2	1.4	0.9	1.2	1.1
		(0.8-1.8)	(0.8-1.8)	(0.8-2.4)	(0.5-1.9)	(0.7-2.0)	(0.5-2.2)
	Daily	**1.6**	**1.5**	**1.8**	1.2	1.4	1.6
		**(1.1-2.4)**	**(1.0-2.2)**	**(1.0-3.0)**	(0.7-2.3)	(0.9-2.3)	(0.8-3.2)
Study school, weekend (≥ 2 hr/day)	Never	1.0	1.0	1.0	1.0	1.0	1.0
	Sometimes	1.4	1.4	1.5	1.1	1.3	1.4
		(0.9-2.0)	(0.9-2.0)	(0.9-2.7)	(0.6-2.1)	(0.7-2.2)	(0.7-2.7)
	Daily	**2.3**	**2.3**	**2.5**	**2.0**	**2.0**	**2.7**
		**(1.6-3.3)**	**(1.5-3.3)**	**(1.5-4.2)**	**(1.1-2.6)**	**(1.2-3.2)**	**(1.4-5.0)**

Bold numbers represents significant result.

† Mixed effect logistic regression was used. Age, gender, school type and grade were adjusted. School was treated as random effect.

‡ Mixed effect logistic regression was used. Age, gender, grade were adjusted. School was treated as random effect.

§ Mixed effect logistic regression was used. Age, school type, grade were adjusted. School was treated as random effect.

Breakfast consumption, whether daily or sometimes, was significantly associated with higher levels of physical activity as compared to never breakfast consumption. Daily breakfast consumers were 2.9 (95% CI: 1.9- 4.4) times more likely to do mild intensity physical activity (≥1/2 hr/day), 2.7 (95% CI: 1.7- 4.1) times more likely to do moderate intensity physical activity (≥1/2 hr/day) and 1.7 (95% CI: 1.2- 2.6) times more likely to do strenuous physical activity (≥1/2 hr/day) than never breakfast consumers. Unlike the dietary behaviors, the odds of engaging in these behaviors were also significantly higher for sometimes breakfast consumers compared to never breakfast consumers. The results were similar among Private school students and boys, while only moderate intensity physical activity (≥1/2 hr/day) was significantly associated with daily breakfast consumption among girls.

Breakfast consumption was not significantly associated with sedentary behaviors such as watching TV/videos or using computer. However, among those who consumed breakfast daily, the odds of studying over weekends (≥2 hr/day) was 2.3 times (95% CI: 1.5-3.3) and in weekdays (≥2 hr/day) it was 1.5 times (95% CI: 1.0- 2.2), compared to never breakfast consumers. No differences between sometimes and never breakfast consumers were observed.

### Psychosocial factors and breakfast consumption

The relationship between psychosocial factors and breakfast consumption is presented in Table 6. Breakfast consumption was positively associated with positive values and beliefs about healthy eating; body image satisfaction; and positive peer and parent influence (p<0.01). In sub-group analyses, peer and parent influence was significantly related to breakfast consumption across all categories (i.e. among both boys and girls, among Private and Government school students). Values and beliefs were significantly associated with breakfast consumption among Government school students and boys only, and body image satisfaction was significantly related to breakfast consumption among Government school students only. Breakfast consumption was inversely associated with depression overall (p<0.01), among Private school students (p<0.05) and among girls (p<0.01).

**Table 6:** Relationship between psychosocial factors and breakfast consumption (n=1814)

**Psychosocial factors**‡	**Overall**	**School Type**		**Gender**	
		**Private**	**Government**	**Boys**	**Girls**
	**Estimate**† **(SE)**	**Estimate**† **(SE)**	**Estimate**† **(SE)**	**Estimate**† **(SE)**	**Estimate**† **(SE)**
**Values**	0.09^**^	0.07	0.11^*^	0.09^*^	0.10
	(0.03)	(0.05)	(0.05)	(0.04)	(0.06)
**Belief**	0.08^**^	0.06	0.11^*^	0.14^**^	−0.02
	(0.03)	(0.04)	(0.05)	(0.04)	(0.06)
**Body Image Satisfaction**	0.07^*^	0.02	0.11^*^	0.05	0.09
	(0.03)	(0.05)	(0.04)	(0.04)	(0.05)
**Depression**	−0.08^**^	−0.10^*^	−0.06	−0.05	−0.14^**^
	(0.03)	(0.05)	(0.04)	(0.04)	(0.05)
**Eating more than usual**	−0.01	0.00	−0.01	−0.00	−0.01
	(0.03)	(0.05)	(0.05)	(0.04)	(0.05)
**Peer’s influence**	0.12^**^	0.13^**^	0.10^*^	0.10^**^	0.01^*^
	(0.32)	(0.05)	(0.05)	(0.04)	(0.06)
**Parents’ Influence**	0.17^**^	0.17^**^	0.17^**^	0.17^**^	0.19^**^
	(0.03)	(0.05)	(0.05)	(0.04)	(0.06)

† Estimates are β coefficients along with standard error obtained from mixed effect regression models. Gender, School type (when not segregated), age, grade and BMI were adjusted and school was treated as random effect. Psychosocial factors are independent and breakfast dependent variable.

‡ Psychosocial factors are standardized scores. A higher score is protective for value, belief, body image satisfaction, peer’s influence and parents influence. A lower score is protective for depression and eating more than usual.

* p<0.05; ** p<0.01.

## Discussion

This study explored the patterns of breakfast consumption among 8^th^ and 10^th^ grade students in Delhi, India and its association with overweight and obesity in this context. Behavioral and psychosocial correlates of breakfast consumption were also evaluated. An important finding was that 30% of these school students in Delhi did not consume breakfast daily. In this study, non-daily consumption of breakfast was greater among higher SES (Private school) students, for whom obesity is most problematic in this context [6,51]. This is troubling, as results from this study highlight that daily breakfast intake is associated with a lower likelihood of overweight and obesity among urban Indian adolescents. This latter finding is consistent with a growing body of literature, worldwide [12-16].

Though earlier studies suggest that regular breakfast consumption is more common among girls in India [34], and among boys in the developed countries [15,52], we did not find significant differences in the frequency of breakfast consumption between boys and girls in the present study. Older students were found to skip breakfast more often in our study (i.e. older age groups and 10^th^ graders as compared to younger age groups and 8^th^ graders), which is consistent with prior literature from developed countries that suggests breakfast skipping increases with age [15,52]. Government schools generally cater to the poor (lower income and lower-middle income class) in Indian context. Students belonging to lower income group are more likely to experience scarcity of good quality food amounting to a lack of wholesome breakfast as well as other meals of the day. While it is known that neither Private nor Government schools in India provide breakfast, a surprising finding in our study was that more Government school students were found to consume breakfast daily as compared with Private school students. The quality of breakfast consumed by Government school students should be explored further to ascertain whether poor quality of breakfast consumed by Government school students or regular breakfast consumption as observed in our study is a reason for Government school students being less overweight and obese, as compared with Private school students in India [6,51,53,54].

Breakfast consumption was found to be associated with overweight and obesity in a dose-dependent manner. That is, regular consumers of breakfast were significantly less overweight and obese as compared to breakfast skippers, who, in turn, were significantly less overweight and obese than never breakfast consumers. Our finding is consistent with several other studies undertaken across the world [11,13-15,18], and suggests that despite the fact that nutritional diversity exists in India, the importance of regular breakfast in maintaining a healthy body weight is not undermined. As this is a cross-sectional study, results might also suggest that those who are overweight and obese try to avoid breakfast in order to skip meals and thereby reduce their weight [55]. Few studies conducted in the West, however, suggest that this inverse relation persists between breakfast consumption and excess weight even longitudinally [12,14]. Such longitudinal studies are yet lacking in India.

The results of this study suggest that as compared to those who skip or never consume breakfast, intake of dairy products, fruits and vegetables is one to five times more among daily breakfast consumers. Thus, daily breakfast consumers have nutritionally better quality of food selection in other meals too as compared to those who skip or never consume breakfast. These findings are similar to research conducted in the West, which suggests that children who consume breakfast make better food choices throughout the day [56,57], such as consumption of more vegetables and less fried foods [58]. Other studies from the developed countries have shown that breakfast skipping, in contrast, is associated with greater consumption of high-fat snacks [59], and skipping of other meals [60], which has the potential to lead to nutritional inadequacy and weight gain.

Among Private school students, non-daily and daily breakfast consumption were significantly associated with ≥ 30 minutes of mild, moderate, and strenuous physical activity levels per day. This finding is consistent with studies undertaken in developed nations [23]. Previous studies have shown that breakfast consumption is positively associated with academic achievement among students [31-33]. Though we did not specifically assess academic achievement as an outcome, we found a positive correlation between breakfast consumption and more study hours especially over weekends across school type and gender. In the Indian context, a positive association of breakfast consumption with physical activity and increased study hours has been demonstrated for the first time through a cross-sectional study. However, further longitudinal studies are required to assess whether the relation is causal or these behaviors simply co-occur.

Psychosocial correlates of breakfast consumption were explored for the first time in the Indian context in this study. Valuing a healthy diet and believing that one’s diet can affect health were associated with breakfast consumption. These findings are in line with earlier studies undertaken in developed countries [28], and are consistent with the theory of reasoned action [61]. The theory of reasoned action would suggest that individual’s beliefs about the benefits associated with the behavior (e.g. regular breakfast consumption would lead to better health) predict the behavioral intentions of the person, which in turn would predict the behavior (daily breakfast consumption). More positive parent and peer influence were consistently associated with breakfast consumption across all sub-groups considered here. These factors, therefore, may be especially important to address in any future intervention that seeks to increase the frequency of breakfast consumption among school-going youth in India. Our study reiterates findings reported in other studies from developed countries [30,62,63], and highlights the importance of parental attitudes and behaviors in creating healthy lifestyle habits such as breakfast consumption in their children as well as the ability of peers to influence dietary behaviors of adolescents. Depression was also shown to be negatively associated with breakfast consumption behavior. Our findings are in line with earlier studies from developed countries which suggest that depression is positively associated with health compromising attitudes and behaviors and negatively associated with health promoting behaviors such as consuming breakfast, lunch and dinner [64].

### Limitations

No objective measures to assess food behavior or physical activity were used in this study. Self-reported data was relied upon, which has the likelihood to be subject to recall bias. We tried to minimize the bias by using a questionnaire which has been validated in earlier large studies in the US [43,44], and extensively pilot tested in India. As this is a cross-sectional study it is important to note that the associations observed here could be bi-directional, with no clear evidence of exposure preceding the outcome. Factors that are associated with breakfast intake could also be associated with overweight/obesity. We did not address the multi-collinearity between breakfast intake and other dietary, physical activity and psychosocial factors while relating breakfast intake to overweight and obesity. It would be interesting to see how these factors mediate the association between breakfast intake and overweight and obesity among Indian adolescents and needs to be explored further. There is a need for longitudinal studies with good controls in India to adequately assess causal relationships like these. Apart from irregular frequency of breakfast consumption, poor quality of breakfast has also been suggested as a risk factor for high BMI levels [65]. In a country such as India with wide socio-economic disparities and cultural as well as nutritional diversity, adequacy of the quality of breakfast contents needs to be explored. This was not within the purview of the current study and should be investigated in future studies.

## Conclusion

Regular breakfast consumption is negatively associated with overweight and obesity among urban school-going adolescents in Delhi. Daily breakfast consumption is also associated with healthier food choices and greater physical activity as compared to never breakfast consumption in this group. Considering these advantages, future interventions to prevent or treat childhood obesity in this context should consider promoting daily breakfast consumption with special focus on Private schools and students from older age groups. There is scope for improvement, as one-third of the students in the present study did not eat breakfast daily. Such programs can include provision of healthy breakfast in schools or having a short break in the morning to allow students to consume breakfast that they can carry from home. Many schools in Delhi start very early in the morning, leaving no time for students to have a wholesome breakfast [34], which is an important barrier to overcome. These interventions should emphasize a supportive social environment to influence parents and peers to promote breakfast consumption, too.

## Competing interests

The author(s) declare that they have no competing interests.

## Authors’ contributions

MA was involved in all aspects including the study concept and design, acquisition, analysis and interpretation of the data, critically revising the manuscript for intellectual consent and giving a final approval to this manuscript. GPN was involved in review of literature, interpretation of the data and drafting the manuscript. VKG was involved in data analysis and drafting the data analysis and results section of the manuscript. CLP and KSR were involved in overall supervision, concept and design and technical guidance throughout the study. MHS provided technical guidance with respect to data analysis and critically revised the manuscript for intellectual content. All authors read and approved the final manuscript.

## Authors’ information

MA (PhD.) is the Director, Health Promotion and Adjunct Assistant Professor at Public Health Foundation of India (PHFI) as well as the Senior Director at Health Related Information Dissemination Amongst Youth (HRIDY), New Delhi; GPN (MBBS; MSc) is the Manager (Research) at HRIDAY, New Delhi; VKG (MPhil.) is the data analyst at PHFI, New Delhi; CLP (PhD.) is Professor & Regional Dean, University of Texas School of Public Health (UTSPH), Austin Regional Campus, Michael & Susan Dell Center for Health Living; KSR (DM) is Professor & President, PHFI, New Delhi; MHS (PhD.) is the Assistant Professor, UTSPH, Austin Regional Campus, Michael & Susan Dell Center for Health Living.

## Pre-publication history

The pre-publication history for this paper can be accessed here:

http://www.biomedcentral.com/1471-2458/12/881/prepub
